# USE OF THE FIBROSIS-4 INDEX FOR SCREENING LIVER FIBROSIS IN PEOPLE LIVING WITH HUMAN IMMUNODEFICIENCY VIRUS AND GLUCOSE METABOLISM DISORDERS

**DOI:** 10.1590/S0004-2803.24612026-003

**Published:** 2026-07-24

**Authors:** Arthur Cavalcante LOPES, Amanda Aymoré SANTOS, Ivo da Cunha NUNES, Jeremias Estevam LOPES, Rosana Maria Feio LIBONATI

**Affiliations:** 1 Universidade Federal do Pará (UFPA), Instituto de Ciências Médicas (ICM), Belém, PA, Brasil.

**Keywords:** HIV, non-alcoholic fatty liver disease, diabetes mellitus, HIV, doença hepática gordurosa não alcoólica, diabetes mellitus

## Abstract

**Background and Objective:**

To evaluate the performance of the Fibrosis-4 (FIB-4) index for liver fibrosis screening in people living with HIV (PLWH) with glucose metabolism disorders (diabetes and prediabetes).

**Methods:**

Cross-sectional study including 100 PLWH on continuous antiretroviral therapy at a reference center in Brazil (March-November 2025). Clinical, laboratory, and ultrasonographic data were collected for FIB-4 calculation. Participants with diabetes were compared to non-diabetic individuals.

**Results:**

Altered FIB-4 values were observed in 32 participants, with no sex differences. Mild steatosis (S≥1) was present in 47.0%, and clinically significant steatosis (S≥2) in 11.0%. S≥1 was associated with higher BMI, waist circumference, visceral fat, total cholesterol, LDL, triglycerides, and ALT levels. FIB-4 showed limited ability to classify S≥2.

**Conclusion:**

FIB-4 is useful for fibrosis screening, particularly to exclude advanced disease, but does not adequately identify clinically significant steatosis (S≥2). Its use should be combined with metabolic assessment and elastography when values are ≥1.3.

## HIGHLIGHTS

FIB-4 is effective as a first-line noninvasive tool to screen liver fibrosis, particularly to rule out advanced disease due to its high negative predictive value.

FIB-4 shows poor performance for detecting clinically significant steatosis (S≥2), with low sensitivity at standard cutoffs.

32% had elevated FIB-4; steatosis prevalence was 47% (S≥1) and 11% (S≥2), with no sex differences.

FIB-4 ≥1.3 should prompt further evaluation with elastography and metabolic assessment, as it is insufficient as a standalone tool for steatosis detection.

## RESUMO

**Contexto e Objetivo:**

Avaliar o risco de fibrose hepática pelo índice Fibrosis-4 (FIB-4) em pessoas vivendo com HIV (PVHIV) com distúrbios do metabolismo glicídico (pré-diabetes e diabetes).

**Métodos:**

Estudo transversal com 100 PVHIV em terapia antirretroviral contínua, atendidas em centro de referência em Belém, Brasil (março-novembro de 2025). Foram coletados dados clínicos, laboratoriais e ultrassonográficos para cálculo do FIB-4. Participantes com diabetes foram comparados aos não diabéticos.

**Resultados:**

Valores alterados de FIB-4 foram observados em 32 participantes, sem diferença entre os sexos. Esteatose leve (S≥1) ocorreu em 47,0% e esteatose clinicamente significativa (S≥2) em 11,0%. S≥1 associou-se a maiores valores de IMC, circunferência abdominal, gordura visceral, colesterol total, LDL, triglicerídeos e ALT. O FIB-4 apresentou capacidade limitada para classificar S≥2.

**Conclusão:**

O FIB-4 é útil para triagem de fibrose hepática, especialmente para excluir doença avançada, mas não identifica adequadamente esteatose clinicamente significativa (S≥2). Deve ser associado à avaliação metabólica e à elastografia quando ≥1,3.

## INTRODUCTION

With the increased life expectancy of people living with HIV (PLWH), it has become evident that chronic HIV infection predisposes individuals to a persistent inflammatory state associated with disturbances in glucose metabolism and insulin resistance^1^. In this context, the liver plays a central role. Metabolic dysfunction-associated steatotic liver disease has become particularly prevalent, ranging from simple steatosis to steatohepatitis with fibrosis, and is driven by obesity, type 2 diabetes, hypertension, and dyslipidemia^2^ ^,^ ^3^.

Biopsy remains the gold standard for assessing inflammation and fibrosis, but routine clinical practice requires feasible noninvasive methods. Elastography and clinical-laboratory scores have gained prominence, such as the Fibrosis-4 (FIB-4) index^4^. The FIB-4 combines age, aspartate aminotransferase (AST), alanine aminotransferase (ALT), and platelet count, showing acceptable performance for screening significant fibrosis^5^. Recent guidelines recommend its use as a first-line approach, particularly in adults with prediabetes or diabetes; values ≥1.3 typically prompt transient elastography, a strategy that correlates with histological findings and helps reduce unnecessary biopsies^6^. In individuals with diabetes, higher FIB-4 scores have been associated with overall mortality, cardiovascular events, carotid intima-media thickness, and chronic kidney disease^7^ ^,^ ^8^. Current international guidelines, including those from the European Association for the Study of the Liver (EASL) and the American Association for the Study of Liver Diseases (AASLD), recommend the use of noninvasive scores such as the FIB-4 index as a first-line screening tool for liver fibrosis, followed by additional assessment in indeterminate or high-risk cases^9^ ^,^ ^10^.

In this context, the present study aims to assess the risk of liver fibrosis in PLWH exhibiting altered glucose metabolism disorders, using the FIB-4 index. Specifically, it seeks to estimate the prevalence of fibrosis risk in this population, characterize the frequency of prediabetes and diabetes, and identify factors associated with elevated FIB-4 scores, focusing on anthropometric, metabolic, and ultrasonographic indicators of steatosis.

## METHODS

This analytical cross-sectional observational study was conducted at the Tropical Medicine Center in Belém, Brazil, between March and November 2025. The target population comprised PLWH receiving regular follow-up at the endocrinology outpatient clinic. A convenience sample of 100 participants was included. The study was initiated following informed consent and approval by the Research Ethics Committee of the Institute of Health Sciences, Núcleo de Medicina Tropical-NMT/Universidade Federal do Pará - UFPA (approval number 7.423.664; CAAE: 85431324.2.0000.5172).

Eligibility criteria were age ≥18 years, continuous use of antiretroviral therapy (ART) for at least 12 months, and willingness to participate in the study. Individuals using chronic corticosteroids or with active opportunistic infections were excluded. The minimum sample size was estimated based on a hepatic fibrosis prevalence of 8.3% with a 95% confidence interval and a maximum error of 5%, resulting in a minimum of 55 participants^11^. The study followed the STROBE guidelines for observational research^12^.

Data were obtained from medical records and routine laboratory and imaging exams. AST, ALT, platelet count, and abdominal ultrasonography with visceral fat measurement (distance between the inner rectus abdominis and anterior aortic wall) performed within six months prior to inclusion were recorded; older or missing exams were repeated. FIB-4 was calculated as (Age×AST) / [Platelets×√(ALT)], with platelets in 10^9^/L. Additional variables included sex, age, ART type and duration, fasting glucose, HbA1c, total cholesterol, LDL, HDL, triglycerides, Body mass index (BMI), and waist circumference. Participants were grouped by type 2 diabetes mellitus status. Data were stored in Microsoft® Excel 2010 and analyzed using BioEstat® 5.3 and Epi Info™ 7.0. Continuous variables were expressed as mean ± SD or median ± IQR; categorical variables as absolute and relative frequencies. 95% CIs were calculated. Normality was assessed by D’Agostino-Pearson test; comparisons used chi-square, Fisher’s exact, t-test, or Mann-Whitney U test, with *P*<0.05.

To evaluate the accuracy of FIB-4 for clinically significant steatosis (S≥2; grade ≥2) on ultrasonography, 2×2 contingency tables were constructed using prespecified cutoffs. For participants younger than 65 years, a FIB-4 cutoff of 1.3 was applied. For those aged 65 years or older, a higher cutoff of 2.0 was used, in accordance with established age-adjusted criteria. These thresholds were selected to improve diagnostic accuracy and minimize misclassification of fibrosis severity. Confusion matrices were reported, including true positives (TP), false positives (FP), true negatives (TN), and false negatives (FN), and sensitivity and specificity with 95%CI were derived using the Wilson method. Analyses were conducted for the overall sample and stratified by glycemic status-normoglycemia versus elevated glycemia (prediabetes + diabetes)-according to the American Diabetes Association (ADA) criteria: diabetes (fasting glucose ≥126 mg/dL and/or HbA1c ≥6.5%) and prediabetes (100-125 mg/dL and/or HbA1c 5.7-6.4%)^13^.

## RESULTS

A total of 100 people living with HIV receiving care at the Tropical Medicine Center were included, with an equal distribution between females (n=50) and males (n=50), as shown in Table 1.

**TABLE 1:** Social demographic characteristics of patients living with HIV attending the Tropical Medicine Center between March and November 2025.

Variable	Female (n=50)	Male (n=50)
Age (years)		
31-39	4 (8.0%)	2 (4.0%)
40-65	36 (72.0%)	35 (70.0%)
66-82	10 (20.0%)	13 (26.0%)
Ethnicity		
Mixed-race	33 (66.0%)	23 (46.0%)
White	9 (18.0%)	16 (32.0%)
Black	8 (16.0%)	10 (20.0%)
Asian	-	1 (2.0%)
Duration of ART (years)		
0-4	7 (14.0%)	1 (2.0%)
5-9	10 (20.0%)	8 (16.0%)
10-14	3 (6.0%)	11 (22.0%)
15-19	11 (22.0%)	7 (14.0%)
20-24	13 (26.0%)	6 (12.0%)
25-29	5 (10.0%)	11 (22.0%)
30-34	1 (2.0%)	6 (12.0%)

Categorical variables are presented as n (%), with percentages calculated relative to the corresponding sex. Value equal to zero, not resulting from rounding.

The overall median FIB-4 value was 1.25 (IQR:0.65). Stratified by sex, the median was 1.19 (IQR:0.53) in women and 1.26 (IQR:0.85) in men, with no statistically significant difference between groups (Mann-Whitney U test, *P*=0.072).

Using an age cutoff of 65 years, the prevalence of altered FIB-4 was 29.9% (23/77) in participants <65 years and 39.1% (9/23) in those ≥65 years (chi-square test, *p*=0.403). Regarding sex, 38 women (76.0%) had normal FIB-4 and 12 (24.0%) had altered FIB-4, whereas 30 men (60.0%) had normal FIB-4 and 20 (40.0%) had altered FIB-4, with no statistically significant association (chi-square test, *P*=0.086). Table 2 presents the distribution of FIB-4 across different glycemic profiles.

**TABLE 2:** FIB-4 in people living with HIV and glucose metabolism disorders attending the Tropical Medicine Center between March and November 2025.

Variable	Normal	Prediabetes	Diabetes	***P*-value**
**Normal FIB-4 (n=68) (%)**	20 (29.4%)	31 (45.6%)	17 (25%)	
**Elevated FIB-4 (n=32) (%)**	5 (15.6%)	16 (50.0%)	11 (34,4%)	0.297^a^

^a^ Chi-square test.

A significant negative correlation was found between FIB-4 and estimated glomerular filtration rate (eGFR) (r= −0.41; *P*<0.001; pearson), indicating that higher FIB-4 values were associated with reduced renal function. Participants with elevated FIB-4 were older (61.0±10.1 vs 56.3±11.1 years; *P*=0.043) and showed lower eGFR (66.4±25.2 vs 76.6±21.1 mL/min/1.73m²; *P*=0.038), higher AST (34.0±18.7 vs 23.0±8.8 U/L; *P*<0.001), higher ALT (31.2±34.7 vs 23.1±17.2 U/L; *P*=0.012), and lower platelet counts (248.5±201.6 vs 332.0±250.8 ×10³/µL; *P*=0.008). BMI (26.1±6.8 vs 26.9±5.9 kg/m²; *P*=0.790), waist circumference (93.0±18.8 vs 91.5±15.0 cm; *P*=0.625), visceral fat (3.8±3.3 vs 4.1±1.6 cm; *P*=0.877), fasting glucose, HbA1c, total cholesterol, LDL, HDL, and triglycerides did not differ between groups. Consistently, no significant correlations were observed between FIB-4 and fasting glucose (r= −0.08; *P*=0.449; spearman), HbA1c (r= −0.02; *P*=0.823; spearman), total cholesterol (r= −0.13; *P*=0.208; spearman), LDL (r= −0.12; *P*=0.233; spearman), HDL (r=0.06; *P*=0.563; pearson), or triglycerides (r= −0.13; *P*=0.189; spearman).

Among the 100 patients evaluated by ultrasonography, the prevalence of mild hepatic steatosis (S≥1) was 47.0% (47; 95%CI 37.5-56.7). Stratified by FIB-4, S≥1 was observed in 45.6% of participants with normal FIB-4 (31/68) and 50.0% of those with altered FIB-4 (16/32), with no significant association (chi-square test, *P*=0.843; OR=0.84; 95%CI 0.36-1.94). Clinically significant steatosis (S≥2) had an overall prevalence of 11.0% (11; 95%CI 6.3-18.6), occurring in 13.2% of participants with normal FIB-4 (9/68) and 6.3% of those with altered FIB-4 (2/32), also without significant association (Fisher’s exact test, *P*=0.495; OR=2.29; 95%CI 0.46-11.27).

The performance of FIB-4 was assessed at prespecified cutoffs of 1.3 (screening) and 2.67 (high risk) for the outcome of clinically significant steatosis (S≥2) on ultrasonography. Table 3 presents the confusion matrices (TP/FP/TN/FN) and the corresponding sensitivity and specificity with 95% confidence intervals for the overall sample (n=100) and for participants with elevated glycemia (prediabetes + diabetes, ADA criteria; n=75).

**TABLE 3:** Performance of FIB-4 for the outcome of clinically significant steatosis in people living with HIV attended at the Tropical Medicine Center between March and November 2025.

Stratum	FIB-4 cutoff	TP/FP/TN/FN	Sensitivity	Specificity
**Overall (n=100)**	1.3	3/41/48/8	27.3% (95%CI 9.7-56.6%)	53.9% (95%CI 43.6-63.9%)
	2.67	0/6/83/11	0.0% (95%CI 0.0-25.9%)	93.3% (95%CI 86.1-96.9%)
**Elevated glycemia** ^a^ **(n=75)**	1.3	3/33/32/7	30.0% (95%CI 10.8-60.3%)	49.2% (95%CI 37.5-61.1%)
	2.67	0/6/59/10	0.0% (95%CI 0.0-27.8%)	90.8% (95%IC 81.3-95.7%)

^a^ Diabetes + Prediabetes.

## DISCUSSION

In this study population, 32% of participants presented with elevated FIB-4, with an equal sex distribution (50% women; 50% men) and no sex differences in continuous FIB-4 values (overall median 1.25; IQR:0.65; women 1.19 [IQR:0.53] vs men 1.26 [IQR:0.85]; Mann-Whitney U, *P*=0.072). Population-based studies have reported lower proportions of elevated FIB-4, such as 9%^14^. In a U.S. cohort, 52.3% maintained normal values over one year^15^, whereas a Japanese cohort reported 78.5%^16^. Among people living with HIV (PLWH) with hepatic steatosis, 72.8% were above the upper limit^17^.

Another finding was the proximity of the median FIB-4 in our cohort to previously published metabolic series. The median FIB-4 in our sample was 1.25 (IQR:0.65), comparable to reported means in type 2 diabetes: 1.15±0.52 and 1.4±0.72^18^ ^,^ ^19^. In HIV-positive women, a median of 0.86 was reported^20^. In men with type 2 diabetes, a mean of 1.64±1.19 was observed^21^. Differences across studies likely reflect the use of different metrics (median vs mean), as well as sample composition, age, and metabolic profile.

Analysis of FIB-4 in relation to anthropometric parameters revealed no significant differences between participants with normal and elevated FIB-4: median BMI was 26.85 kg/m² in the normal group versus 26.06 kg/m² in the elevated group (*P*=0.790); waist circumference 91.5 cm vs 93.0 cm (*P*=0.625); and visceral fat on ultrasonography 4.10 cm vs 3.80 cm (*P*=0.877). These findings are consistent with literature reporting modest or inconsistent associations between adiposity and FIB-4 in metabolic contexts. A negative correlation between BMI and FIB-4 was described in individuals with insulin resistance without diabetes^22^. Similar FIB-4 means across BMI strata (<25 and ≥25 kg/m²) in diabetes were also observed^23^.

Regarding glycemic profiles, no statistically significant association was observed between elevated FIB-4 and prediabetes (OR 1.19; 95%CI 0.51-2.77; *P*=0.680), diabetes (OR 1.57; 95%CI 0.63-3.92; *P*=0.330), or elevated glycemia (prediabetes + diabetes; OR 2.25; 95%CI 0.76-6.68; *P*=0.137), nor with fasting glucose (r= −0.08; *P*=0.449) or HbA1c (r= −0.02; *P*=0.823). These results align with metabolic series where central FIB-4 values in type 2 diabetes were modest and similar to ours: 1.15±0.5 and 1.4±0.7 vs 1.25 in the present sample^18^ ^,^ ^19^.

Ultrasonography revealed a prevalence of 47.0% for mild hepatic steatosis (S≥1) and 11.0% for clinically significant steatosis (S≥2), with no sex differences. Participants with S≥1 had higher BMI (28.4±5.9 vs 25.5±3.8), waist circumference (95.4±12.6 vs 88.7±11.2), visceral fat (4.7±1.8 vs 3.9±1.4), total cholesterol (170.0 vs 148.0 mg/dL), LDL (95.0 vs 77.0 mg/dL), triglycerides (150.0 vs 121.0 mg/dL), and ALT (31.9±29.6 vs 24.0±11.5), all *P*<0.05. HDL, eGFR, fasting glucose (106.0±45.5 vs 96.0±19.0), and HbA1c (6.0±1.0 vs 5.9±0.7) did not differ (*P*>0.05). Continuous FIB-4 values were similar across steatosis strata (1.3±0.7 vs 1.2±0.6; *P*=0.820), reinforcing its utility primarily for fibrosis screening rather than for diagnosing steatosis^22^. In PLWH, lower imaging-based prevalences have been reported, highlighting clinical heterogeneity^17^. In the present study, the prevalence of S≥1 was 47.0%.

At prespecified cutoffs (Table 3), FIB-4 showed limited performance in classifying S≥2. In the overall sample, at 1.3, sensitivity was 27.3% (95%CI 9.7-56.6) and specificity 53.9% (43.6-63.9), with a negative predictive value of 85.7% (74.3-92.6) in a low-prevalence setting (S≥2: 11.0%). At 2.67, sensitivity decreased to 0.0% (0.0-25.9) while specificity increased to 93.3% (86.1-96.9). A similar pattern was observed among participants with elevated glycemia (1.3: 30.0% and 49.2%; 2.67: 0.0% and 90.8%, for sensitivity and specificity, respectively). Likelihood ratios close to 1 indicate limited reclassification ability, consistent with evidence that FIB-4 is more effective for excluding advanced fibrosis than for detecting clinically significant steatosis^24^.

## CONCLUSION

Elevated FIB-4 values were observed in 32% of participants, with no sex differences. Steatosis prevalence was 47.0% for S≥1 and 11.0% for S≥2; S≥1 was associated with higher BMI, waist circumference, visceral fat, total cholesterol, LDL, triglycerides, and ALT, while HDL, eGFR, fasting glucose, and HbA1c did not differ. FIB-4 remains useful for fibrosis screening, particularly to exclude advanced disease, but does not replace methods for quantifying hepatic fat when identifying S≥2; in practice, its use should be integrated with metabolic assessment and elastography when FIB-4 ≥1.3. Study limitations include its cross-sectional design, convenience sampling, few S≥2 events (n=11), ultrasound-based outcomes without systematic elastography or histology, and single-center setting, which limits generalizability and underscores the need for larger longitudinal studies.

## Data Availability

Data in article: the research data are presented within the article itself (available in Tables 1, 2 and 3).
